# Editorial: Advances in therapeutic drug monitoring of psychiatric subjects: Analytical strategies and clinical approaches

**DOI:** 10.3389/fpsyt.2022.1056380

**Published:** 2022-10-18

**Authors:** Laura Mercolini

**Affiliations:** Research Group of Pharmaco-Toxicological Analysis (PTA Lab), Department of Pharmacy and Biotechnology (FaBiT), Alma Mater Studiorum—University of Bologna, Bologna, Italy

**Keywords:** analytical strategies, pharmaco-toxicological analysis, chemical-clinical correlations, therapeutic drug monitoring (TDM), therapeutic reference ranges, treatment adherence

Despite the long and sometimes enthusing history of pharmacological therapy in psychiatry, the hard truth is that unfortunately a quite large percentage of patients is still not responding, or poorly responding, to treatment, leading to many life years lost to disability, many lost lives, and an immeasurable amount of suffering from patients, relatives, friends, and caregivers alike (1). Thus, any scientific advance and any practice that could lead to even a slight increase in psychiatric therapy effectiveness would also bring with them enormous benefits for both citizens and healthcare institutions. It is a strong conviction, after many years of practice and study, that therapeutic drug monitoring (TDM) is one of these practices, one that is continually advancing and progressing both from the analytical and clinical points of view, toward the final goal of better, personalized, precision medicine (2–7).

TDM is based on the central “dogma” of the existence of chemical-clinical correlations (CCC); that is, the establishment in most patients, at steady-state conditions, of fixed, or at least tight, relationships between:

- the administered dose of a drug and its plasma levels (and/or its metabolites)
- the plasma levels of a drug (and/or its metabolites) and the therapeutic effect(s)
- the plasma levels of a drug (and/or its metabolites) and the toxic (and possibly side) effects.

In the clinical practice, this often corresponds to the determination of therapeutic and toxic reference ranges of drug plasma levels, for each individual drug (8, 9). These are very attractive premises, since the concrete existence of CCC, or of any close approximation of them, would easily allow clinicians to obtain maximum therapeutic effectiveness (i.e., the highest possible percentage of therapeutic success with the least possible percentage and severity of unwanted effects) by adjusting drug doses and dosage intervals according to the results of accurate substance determination in patient biological fluids. This introduces a supplemental element of objective data that can give support and evidence on which to base the therapeutic decisions (2, 10).

It is thus clear that, in exchange for a moderate increase in the total cost of treatment (due to the programmed and repeated drug level determination procedures), TDM can provide much more substantial benefits, in terms of lower hospitalization costs, lower costs for additional therapies (11–13) and, what is more important, better quality of life for the patients and their caregivers.

In this Research Topic, current uses and future perspectives of TDM are considered and discussed, with an interesting mix of different points of view, ranging from medicinal and pharmaco-toxicological chemistry to systematic reviews of the literature, to animal studies and treatment adherence evaluation.

Hart et al. (“*Therapeutic reference ranges for psychotropic drugs: A protocol for systematic reviews*”) have devised an innovative, reliable and systematic approach for the evaluation of existing literature in the determination of population-based reference ranges for drug levels (i.e., for the correct establishment of CCC) in psychiatry. This approach represents an interesting advancement in comparison to existing ones, where CCC are often based on anecdotic data and expert recommendations, which are of course exposed to problems of unreliability and non-standardization due to their non-systematic nature.

Marasca et al. (“*Dried volumetric microsampling approaches for the therapeutic drug monitoring of psychiatric patients undergoing clozapine treatment*”) describe a specific application—the TDM of the atypical antipsychotic clozapine—which is naturally narrow in scope, but provides interesting insight into the possible advantages of sample miniaturization. When correctly implemented, dried microsampling provides substantial savings in the analytical step, thus further increasing the economic advantages of TDM, while also providing better analytical performance due to increased analyte stability (14–17).

Two papers explore the possible role of TDM in the treatment of depression. In fact, TDM application to antidepressants is much less widespread than its application to other psychiatric drugs, such as antipsychotics. Probably, the major hurdle toward a larger TDM application has been the lack of reliable CCC, with most existing studies reporting ambiguous associations between concentration and clinical effect for most antidepressants. Funk et al. (“*Is therapeutic drug monitoring relevant for antidepressant drug therapy? Implications from a systematic review and meta-analysis with focus on moderating factors*”) explore existing literature in an attempt to clarify the true origin of this ambiguity. They hypothesize that methodological shortcomings in clinical studies, rather than intrinsic antidepressant drug peculiarities, could be responsible for this situation, and carry out a systematic review and meta-analysis to verify their hypothesis. Piacentino et al. (“*Therapeutic drug monitoring of antidepressants: An underused but potentially valuable tool in primary care*”), on the other hand, explore the current situation as regards the TDM of antidepressants in the specific setting of primary care. This peculiar point of view is especially significant, since in the primary care setting the indirect cost-effectiveness of TDM can be even more problematic than in specialistic and hospital settings. A case series on the TDM of patients treated with escitalopram highlights the results and the potential benefits of TDM application.

Tao et al. (“*Is aripiprazole similar to quetiapine for treatment of bipolar depression? Results from meta-analysis of Chinese data*”) present a meta-analysis of existing data for the comparison of the effectiveness of aripiprazole and the current reference antipsychotic drug, i.e., quetiapine, in the treatment of bipolar disorder. The role of aripiprazole in bipolar disorder treatment is still unclear, with contrasting data on its efficacy and safety for this specific indication; the paper by Tao et al. can bring more light to this hotly debated subject.

Aguglia et al. (“*The role of attitudes toward medication and treatment adherence in the clinical response to LAIs: Findings from the STAR Network Depot Study*”) brief us on the results of STAR Network's naturalistic, multicentre, observational, prospective “Depot Study” on how patient attitude can influence and to some extent predict treatment outcomes in long-acting injectable (LAI) antipsychotic therapy. The importance of a subjective psychological element in pharmacological psychiatric treatment, and in particular in LAI treatment (18), highlights the need to consider each patient as an individual, thus further reinforcing the usefulness of TDM as a tool for therapy personalisation and optimisation and for increasing patient adherence and trust in their caregivers.

Finally, Zhang et al. (“*5:2 intermittent fasting tapers food intake in the refeeding state and ameliorates metabolic disturbances in mice exposed to olanzapine*”) report the results of an animal study that seeks to clarify the possible usefulness of non-pharmacological treatments in contrasting the onset or the progression of the metabolic syndrome, which represents one of the most frequent severe side effects of olanzapine treatment—and of most atypical antipsychotics.

We hope that this Research Topic will shed some light on the intricacies of TDM application for the optimisation of psychoactive drug treatments, and that it can contribute to bring about significant improvements to patients' wellbeing, however small they can be.

## Author contributions

The author confirms being the sole contributor of this work and has approved it for publication.

## Conflict of interest

The author declares that the research was conducted in the absence of any commercial or financial relationships that could be construed as a potential conflict of interest.

## Publisher's note

All claims expressed in this article are solely those of the authors and do not necessarily represent those of their affiliated organizations, or those of the publisher, the editors and the reviewers. Any product that may be evaluated in this article, or claim that may be made by its manufacturer, is not guaranteed or endorsed by the publisher.
